# Nutritional Status, Breastfeeding, and Evolution of Infants with Acute Viral Bronchiolitis

**Published:** 2007-09

**Authors:** Cristina T.L. Dornelles, Jefferson P. Piva, Paulo J.C. Marostica

**Affiliations:** 1 Nutrition Departament, Hospital de Clínicas de Porto Alegre/RS, Rua: Ramiro Barcelos, 2350, 90035903-Porto Alegre-RS, Brazil; 2 Pediatric Emergency Section; 3 Pediatric and Puericulture Department, Medical School of Universidade Federal do Rio Grande do Sul, Brazil

**Keywords:** Breastfeeding, Bronchiolitis, Infant, Infant nutritional status, Prospective studies, Risk factors, Brazil

## Abstract

Acute viral bronchiolitis is a common respiratory infectious disease of infancy. A prospective study was carried out with 175 infants aged up to six months to evaluate their nutritional and breastfeeding status as possible risk factors for unfavourable evolution of previously-healthy infants from a care hospital. Immunofluorescence test for virus and anthropometric assessment were performed. Outcomes were length of oxygen-use, length of hospital stay, and type of hospital unit needed. Seventy-three percent of the infants were well-nourished, 6% undernourished, 8.6% at a nutritional risk, 10.9% overweight, and 1.7% obese. Eighty-one percent of the undernourished and nutritionally at-risk infants and 72% of the well-nourished, overweight, and obese infants did not receive exclusive breastfeeding. The median length of hospital stay was four days and of oxygen-use was 60 hours. The nutritional status did not affect the clinical course of previously-healthy infants with acute viral brochiolitis. The duration of exclusive breastfeeding, but not type of breastfeeding, was inversely related to the length of oxygen-use and the length of hospital stay. Shorter exclusive breastfeeding was observed in infants who were assigned to a paediatric ward or to an intensive care unit. In conclusion, longer duration of breastfeeding was associated with better clinical outcomes.

## INTRODUCTION

Acute viral bronchiolitis is a common infectious disease of the lower small airways that affects mostly infants aged less than one year (1). Approximately 2.2 cases of acute viral bronchiolitis occur per 100 infants annually, and 1% of these are hospitalized (2). The disease is characterized by a diffuse bronchiolar inflammation induced by viruses (respiratory syncytial virus—responsible for 60–90% of cases, parainfluenza, influenza, rhinovirus, adenovirus, human metapneumovirus, coronavirus, enterovirus, and others) (2-4).

Malnutrition and infection are among the most frequent causes of morbidity and mortality in infancy, especially in developing countries, where the frequency, duration, and severity of the infection are related to the nutritional status of children (5,6).

Breastfeeding provides protection against infections in newborns and infants, and it is associated with low levels of morbidity and mortality in developing countries (7-9). This effect can substantially decrease when the child is fed other than maternal milk, including even water or tea. The reason for this is that the child who is not exclusively breastfed receives less protection factors that exist in mother's milk, besides receiving food or water that are frequently contaminated (9-11).

The present study is original in evaluating such an association in the evolution of acute viral bronchiolitis. Its importance is the impact of the nutritional and breastfeeding status on this infection which has not yet been completely studied. This understanding would lead to the accomplishment of other studies focused on the prevention of malnutrition and breastfeeding interruption in infants with acute viral bronchiolitis. The objective of the present study was to evaluate the risk factors—especially the nutritional status and type of breastfeeding—in the evolution of bronquiolite viral aguda in previously-healthy infants aged up to six months.

## MATERIALS AND METHODS

A prospective transversal study was carried out with previously-healthy infants aged 0–6 month(s), with a clinical diagnosis of acute viral bronchiolitis and first episode of wheezing, who were admitted to the emergency ward or to intensive care paediatric units, in a tertiary public hospital—Hospital de Clínicas de Porto Alegre (HCPA)—in the state of Rio Grande do Sul, in Southern Brazil. The Ethics and Research Committee of this institution approved the study, which was carried out during April 2004–April 2005.

Infants were selected when they arrived at the hospital and were diagnosed by the on-duty emergency paediatrician as having acute viral bronchiolitis based on the symptoms, such as respiratory distress, tachypnea, wheezing, or crackles in pulmonary auscultation, and coughing with a history of upper airway infection with signs and symptoms no longer than seven days.

Prematurely-born (<37 weeks) or low-birthweight (<2,500 g) infants, those with congenital malformations, inborn errors of metabolism, heart, neurological or liver disease, other chronic or intercurrent respiratory diseases, immunodepression, HIV-positive mother, and diseases that could influence the nutritional status (pathologic gastroesophageal reflux, acute gastroenteritis, diarrhoea, or others) were excluded. Although malnutrition could be considered a chronic disease, it was not listed as a criterion for exclusion since, as we will present later on, this was one of the study factors.

Nasal aspirate from the nasopharynx was collected to detect virus by the indirect immunofluorescence test as part of the paediatric emergency routine care of HCPA. The viruses tested were respiratory syncytial virus, parainfluenza, influenza, and adenovirus. The paediatricians were not blind to any data, and the researchers did not influence the management of patients.

If the infant met the inclusion criteria and the parents signed the consent term, a form was filled in with clinical evaluation and with data from the patient records. Data on type of breastfeeding, duration of exclusive breastfeeding, exposure to a smoking mother or passive smoking, i.e. smoking people living in the same home, and socioeconomic situation of the infant's family were collected through a structured interview at admission with parents. Immediately after the infants were weighed and measured by a clinical dietitian and three trained nutrition students, a research nutritionist checked the assessments.

Weight of the infant was measured without clothes on, using a Urano ¯ electronic scale that stands a maximum weight of 15 kg and 5 g precision. The measurement of length was done from the top of the head to the heels with the child lying on a wood slab with a fixed piece on one side and a moveable one on the other (top of head on the fixed part).

Data were processed using the Epi Info^™^ software (version 6.0) (Atlanta, Georgia) that uses the standards from the National Center for Health Statistics (NCHS) as a reference and calculates mean deviations, percentiles, and z-scores for the ratio length-for-age, weight-for-length, and weight-for-age (12).

For classifying the nutritional status of infants, we compared measurements of length-for-age and weight-for-length with the NCHS standard reference. Undernourishment was defined based on the z-score for length-for-age and weight-for-length below −2.00 (<3^rd^ percentile). For obesity, we used the weight-for-length ratio above +2.00 (>97^th^ percentile) according to the criteria recommended by the World Health Organization (WHO) (13,14). For classification of nutritional risk, we considered the z-score levels to be ≤−1.28 (<10^th^ percentile) for weight-for-length, and for overweight a z-score of ≥+1.28 (>90^th^ percentile) for weight-for-length, as recommended by the American Society for Parenteral and Enteral Nutrition (15). The use of the 10^th^ percentile was also adopted for weight-for-age in the classification of the nutritional risk (16). The infants who presented a z-score on the limit between −1.27 and +1.27 for weight-for-length were considered well-nourished. In the classification of nutritional status, where more than one of the anthropometrical ratios was used (length-for-age, weight-for-age, weight-for-length) and a discrepancy existed between the levels used, the lowest one was considered.

In the classification of types of breastfeeding, the categories defined by WHO were adopted (17): Exclusive breastfeeding was considered when the infant received only maternal milk, either directly from the breast or extracted and no other liquid or solid, with the exception of drops or syrups of vitamins, minerals, and/or medicine. Predominant breastfeeding referred to when the infant received together with maternal milk water or water-based drinks, such as fruit juices and tea. The classification of breastfeeding was given when the infant received maternal milk, directly from the breast or extracted, independently of receiving other foods or liquids, including non-human milk. Artificial feeding was considered when the infant was no longer breastfed but received cow's milk—in natural or powdered—and formula feeding for the first semester along with other liquids and solids.

All the infants were monitored until discharge to obtain data with regard to the length of oxygen-use and hospital stay. Clinical data were confirmed by a cross-check with the electronic records after discharge from the hospital. Infants who stayed in the hospital for more than 14 days were excluded.

At the HCPA, the usual criteria for withdrawal of oxygen is the absence of respiratory distress and haemoglobin saturation of >94% in room air. This decision, and that of discharging the patients, was taken by the paediatrician-in-charge.

The clinical evolution of infants with acute viral bronchiolitis was also evaluated according to the more complex hospital unit needed. For such classification, paediatric emergency care was considered as of least complexity where the infants were usually admitted for observation and the intensive care unit as the most complex one. The paediatric ward, in this context, was considered intermediate complexity between the other two.

### Statistical analysis

To calculate the sample size, a preliminary analysis was used considering an α=0.05 and a β=0.20, with a prevalence of 15% of grouped malnutrition and nutritional risk. Considering relevant, a difference of two days of length of hospital stay with standard deviation (SD) of 3.2 days, the need of 145 infants was estimated.

In the association between the quantitative variables in relation to the length of hospital stay and oxygen-use, the Spearman's correlation coefficient was used because the dependent variables presented an asymmetric distribution. To compare the types of breastfeeding and the nutritional classification in relation to the length of hospital stay and oxygen-use, the Mann-Whitney test was used. For the variables with more than two categories, a Kruskal-Wallis test was used, and the Dunn test was run as a complement. Later, a multiple linear regression test was applied.

In the association between the categorical variables and the hospital unit needed or saturation interval, Pearson's chi-square test was applied and, as a complement, the adjusted standardized residual was used for checking local associations.

To compare the quantitative variables in relation to the hospital unit used, either ANOVA or the Kruskal-Wallis test was used, complemented by the Tukey test in the presence of the significant statistical difference and simple and multiple Poisson Regression with over dispersion.

The p value of 0.05 was considered significant, and the analyses were carried out using the SPSS software (version 10.0) (SPSS Inc., Chicago, IL).

## RESULTS

For this study, 190 infants who met the inclusion criteria were enrolled. There were no refusals to participate. Of the 190 infants, 15 were excluded because the length of their stay in the hospital was more than 14 days, which left 175 infants at the time of conclusion. The evaluation of all the 190 infants did not show significantly different results compared to those of the remaining 175 when comparing the nutritional status and types of breastfeeding with the outcomes considered. Data from these 175 infants are presented below.

Table 1 describes the general characteristics of the infants with acute viral bronchiolitis before hospital admission. Of the cases presented, 51.4% of the hospitalized infants evaluated were aged three months or less. Only 1.7% of the infants attended daycare centres. 11.4% of the children had the exposure to a smoking mother and 66.7% to passive smoking—both the factors previously associated with a greater risk for hospitalization due to acute viral bronchiolitis. The data relating to the socioeconomic situation of the infants’ family showed that 44% of mothers and 43.5% of fathers had not completed elementary school. The median monthly family income was € 227 which corresponds to 2.3 minimum national salaries. Initial saturation at admission was >94% in 71.3% of the infants.

**Table 1:** General characteristics of infants with acute viral bronchiolitis at admission

Characteristic	Infants (n=175)	Confidence interval 95%
Age (months)∗	3.2±1.7	2.9–3.4
Sex		
Male, no. (%)	102 (58.3)	57.7–58.9
Female, no. (%)	73 (41.7)	41.1–42.3
Days of symptoms†	3.0 (2–4)	
Initial saturation∗	95.3±3.2	94.8–95.8

∗ Mean±standard deviation;

† Median (25th to 75th)

Results of viral identification tests from nasopharynx secretion showed that 50.3% were respiratory syncytial virus, 4% parainfluenza 3, 1.7% influenza A, 0.6% adenovirus; 36.6% were negative; and samples could not be collected from 6.8% for operational difficulties, like unavailability of reagents during weekends or scarcity of nasal secretions.

Table 2 shows the classification categories of the nutritional status and breastfeeding. The nutritional status was compared in relation to exclusive breastfeeding. Eighty-one percent of the undernourished and nutritionally at-risk infants and 72% of the well-nourished, overweight, and obese infants did not receive exclusive breastfeeding (p=0.009). The other comparisons were not significant.

**Table 2:** Classification of nutritional status and breastfeeding

Characteristics	Infants (n=175)		Confidence interveral 95%
	No.	%	
Z-score of L/A, no. (%)			
<−2.00	7	4	1.8–8.4
−1.99 to −1.29∗	25	14.3	9.6–20.6
−1.28 to +1.28∗	136	77.7	70.7–83.5
+1.29 to +1.99∗	6	3.4	1.4–7.7
<+2.00	1	0.6	0.03–3.6
Z-score of W/A, no. (%)			
>−2.00	4	2.3	0.7–6.1
−1.99 to −1.29	13	7.4	4.2–12.6
−1.28 to +1.28	142	81.1	74.4–86.5
+1.29 to +1.99	15	8.6	5.0–14.0
<+2.00	1	0.6	0.03–3.6
Z-score of W/L, no. (%)			
>−2.00	2	1.1	0.2–4.5
−1.29 to −199	4	2.3	0.7–6.1
−1.28 to +1.28	146	83.4	76.9–88.5
+1.29 to +1.99	19	10.9	6.8–16.7
<+2.00	3	1.7	0.4–5.3
Classification of nutritional status, no. (%)			
Undernourished	11	6.3	3.3–11.3
Nutritional risk	15	8.6	5.0–14.0
Well-nourished	127	72.6	65.2–78.9
Overweight	19	10.9	6.8–16.7
Obese	3	1.7	0.4–5.3
Breastfeed, no. (%)			
Yes	164	94.3	94.0–95.6
No	10	5.7	5.4–6.0
Breastfeeding status, no. (%)			
Exclusive breastfeeding	46	26.3	25.8–26.8
Predominant breastfeeding	20	11.4	11.0–11.8
Breastfeeding	51	29.1	28.6–29.6
Artificial feeding	58	33.1	32.6–33.6
Duration of exclusive breastfeeding (days)	43±41		36.6–48.8

∗ L/A cross-section only used for classification of undernourished infants; L/A=Length-for-age; W/A=Weight-for-age; W/L=Weight-for-length,

The clinical evolution of the infants with acute viral bronchiolitis was evaluated according to the more complex hospital unit needed, length of hospital stay, and oxygen-use. Ninety-three (53.1%) infants needed to be hospitalized in a paediatric ward, but only four (2.3%) were sent to an intensive care unit. The median duration of hospital stay was four days, the initial saturation was 96%, and the length of oxygen-use was 60 hours. There were no deaths among the infants followed.

Sixty-seven percent of the infants in whom the presence of infecting virus was identified needed to be hospitalized in the paediatric ward or intensive care unit compared to 46% of the others who had no infecting virus (p=0.005). Infants admitted to paediatric emergency care had been on exclusive breastfeeding for a median of 45 days compared to 30 days in the paediatric ward and 15 days in the intensive care unit (^2^=6.434; p=0.04). Even when the hospital unit was needed and the presence of virus, age, and nutritional status were analyzed together under a multiple linear regression, the presence of virus and the duration of exclusive breastfeeding continued to present as the only significant correlations. The chance of hospitalization increased by 50% when the virus was identified (relative risk=1.5; confidence interval 1.1–2.0).

Table 3 shows the association between the anthropometric variables studied and the length of hospital stay and oxygen-use. An inverse association was observed among age, weight-for-length and weight-for-age ratios, duration of exclusive breastfeeding, and hours on oxygen-use. A negative correlation between age and length of hospital stay was also observed. Statistically significant correlations were not found between the outcomes evaluated and gestational age, weight, and length at birth, initial saturation, number of rooms and people living in them, family income, and z-score for length-for-age. The same analysis, taking into consideration only the infants in whom any virus or respiratory syncytial virus was identified, was significant in the same correlations presented here.

**Table 3:** Correlations between the variables studied and the length of hospital stay and oxygen-use

Variable (n=175)	Hospital stay		Oxygen-use	
	r∗	p value	r∗	p value
Age	-0.254	0.001	−0.222	0.003
Z-score (L/A)	−0.008	0.913	−0.004	0.954
Z–score (W/L)	−0.107	0.159	−0.164	0.030
Z–score (W/A)	−0.136	0.072	−0.168	0.026
Duration (days) of EB	−0,265	<0,001	−0,333	<0,001

∗ Spearman's correlation coefficient; EB=Exclusive breastfeeding; L/A=Length-for-age; W/A=Weight-for-age; W/L=Weight-for-length

A multiple linear regression was elaborated to evaluate the variables in this study that had significance in the prediction of the length of oxygen-use and of hospital stay. The linear regression model for the variable—length of oxygen-use—was statistically significant by means of multiple regression variance analysis (F_6_, _155_=4.021; p=0.001). As shown in Table 4, the significant predictor values are shown to be duration of exclusive breastfeeding and identification of the virus in the infants studied. Using beta-coefficients of linear regression, it can be interpreted that infants with an identified virus had increased the length of oxygen-use by an average of approximately 24 hours. Since the duration of exclusive breastfeeding variable was measured in days, for each additional day of exclusive breastfeeding, the infants had an average reduction of 0.374 hours in the length of oxygen-use. So, for each month of exclusive breastfeeding, the infants studied presented a reduction of approximately 11 hours in this variable. Likewise, the linear regression model for the variable—length of hospital stay—was statistically significant by means of multiple regression variance analysis (F_3_, _159_=6.512; p<0.001). The duration of exclusive breastfeeding (p=0.018) and the identification of the virus (p=0.018) were the significant predictor values.

**Table 4:** Multiple linear regression for hours of using oxygen

Variable	Linear regression model	
	β	p value
Age (days)	−0.074	0.511
Z-score (W/A)	3.107	0.665
Z-score (W/L)	−5.030	0.482
Nutritional status	−12.931	0.333
Duration (days) of EB	−0.374	0.011
Presence of virus	23.268	0.023

EB=Exclusive breastfeeding; W/A=Weight-for-age; W/L=Weight-for-length

Statistically significant differences were not observed in relation to the length of hospital stay when comparing sex, initial saturation, classification of nutritional status, classification of length-for-age, weight-for-age, and weight-for-length, type of breastfeeding, level of parental education, whether mother was a smoker, and if any of the people living in the home smoked.

The infants with positive virus stayed for an average of 4.8 (±3) days in the hospital, while others stayed for 3.6 (±3.2) days (p=0.004).

## DISCUSSION

In the present study, the nutritional status and type of breastfeeding did not affect the clinical course of previously-healthy infants with acute viral bronchiolitis. On the other hand, the duration of exclusive breastfeeding was inversely related to the length of oxygen-use and the length of hospital stay. Also, infants who needed a less complex care at paediatric emergency had been on exclusive breastfeeding for a longer period than infants assigned to different units.

The study was carried out during the one-year period at a public university hospital evaluating a representative number of 190 infants. The decision to restrict the inclusion of patients to the first six months of life was made because, during this age-range, the diagnosis of acute viral bronchiolitis in infants that present initial acute wheezing is the most probable and not asthma or reactive airway disease. Furthermore, it is during this age that exclusive breastfeeding is recommended, which makes it possible to compare it with other types of feedings that are considered to be less adequate (11,17,18).

The exclusion of 15 infants with more than 14 days of hospitalization represented less than 10% of the sample. The reason these infants were excluded was the possibility of other undetected associated co-morbidity factors since acute viral bronchiolitis is a viral infection that usually has an activity period of 1–2 week(s) (19,20).

Fifty-eight percent of the infants were males, possibly because of smaller airways present in boys. These results are similar to those reported by others (21-23).

The role of infections is very important in the evolution and survival of the malnourished child. The effects of malnourishment and infection, even in the mild and moderate stages, are not additive but multiplicative (24). We did not evaluate the impact of infection on the nutritional status since the study was transversal, but we proposed to check the impact of nutritional status on the clinical evolution of acute viral bronchiolitis.

In different studies, a strong association was evident for the protection of exclusive or predominant breastfeeding against respiratory morbidity as opposed to the introduction of formula milk (8,9,25–27). In agreement with this, in the present study, the longer duration of exclusive breastfeeding was associated with the shorter length of hospital stay and oxygen-use. Breastfeeding for less than one month increased the incidence of respiratory syncytial virus-associated infection. It is reasonable to speculate that human milk may confer several effects on the development of the respiratory tract and its subsequent ability to fight infections and illnesses. The specific nutritional immunoregulatory and immunomodulatory factors in maternal milk may promote maturation of the infant's immune competence (26,27). The optimal duration of exclusive breastfeeding recommended by WHO is six months (18,28–30). Given this recommendation, it is important to stress the role of exclusive breastfeeding in the prevention of childhood illnesses and infection in infants. In Brazil, the most recent poll showed that 96% of women initiated breastfeeding, and only 11% exclusively breastfed during 4–6 months of life, and the mean duration of exclusive breastfeeding was only one month (11).

In relation to the length of oxygen-use and the length of hospital stay, the duration of exclusive breastfeeding and the presence of virus were determinants in the clinical evolution. As for the treatment-location, 67% of the infants identified as having the virus were hospitalized in the paediatric ward or in the intensive care unit, compared to 45% of the others who had no infecting virus. Although this difference could be secondary to a greater availability of beds for infants with an identified virus in the endemic months, these infants also presented other criteria of severity mentioned above. These findings suggest that infants with respiratory syncytial virus and those with shorter duration of exclusive breastfeeding present more severe clinical outcomes (31).

In the present study, a prevalence of undernourishment of 4%, 2.3%, and 1.1% according to the ratios of length-for-age, weight-for-age, and weight-for-length respectively was found, which are satisfactory rates according to the recommendations of WHO and lower when compared with findings of other studies (23,32,33).

No statistically significant associations were found among the anthropometric rates (length-for-age, weight-for-age, and weight-for-length), nutritional status, or among the types of breastfeeding, with the length of oxygen-use, and the length of hospital stay. However, only 11 (6.3%) infants were undernourished, and 15 were at a nutritional risk. It is possible that, with a sample with a greater number of malnourished infants, some difference in the outcomes could be demonstrable.

This study was limited because it evaluated the infants in a hospital setting, i.e. it included only the more severe cases. The study design used could not exclude that a larger proportion of infants from the same population of origin on exclusive breastfeeding had milder cases of bronquiolite viral aguda or even did not get sick, representing a protective effect of the mother's milk, that would not be detected. Another limitation was that the paediatricians were not blind either to the age or to viral diagnosis. This fact could explain, at least in part, the prolonged care taken with infants who were respiratory syncytial virus-positive as this is a known factor of worse prognosis in acute viral bronchiolitis. Social factors may have been involved at the length of hospital stay in some infants.

In conclusion, the shorter duration of exclusive breastfeeding was a risk factor for the unfavourable clinical evolution of acute viral bronchiolitis in previously-healthy infants from a tertiary-care hospital. Shorter exclusive breastfeeding was observed in infants who were assigned to the paediatric ward or to an intensive care unit. These findings emphasize the importance of promoting exclusive breastfeeding up to six months of life, not only because of prevention of infectious diseases but also because of the lesser aggressive course of bronchiolitis in breastfed children.

## References

[1] Denny FW, Clyde WA (1986). Acute lower respiratory tract infections in nonhospitalized infants. J Pediatr.

[2] Wright RB, Pomerantz WJ, Luria JW (2002). New approaches to respiratory infections in infants. Bronchiolitis and croup. Emerg Med Clin North Am.

[3] Ebihara T, Endo R, Kikuta H, Ishiguro N, Ishiko H, Kobayashi K (2004). Comparison of the seroprevalence of human metapneumovirus and human respiratory syncytial virus. J Med Virol.

[4] Pitrez PM, Stein RT, Stuermer L, Macedo IS, Schmitt VM, Jones MH (2005). [Rhinovirus and acute bronchiolitis in young infants]. J Pediatr (Rio J).

[5] Lesourd BM, Mazari L (1997). Immune responses during recovery from protein-energy malnutrition. Clin Nutr.

[6] (1998). Organização Pan-Americana de Saúde-Opas. Atencion integrada a las enfermidades prevalente e la infância (AIEPI) em las Américas. Bol Epidemiol.

[7] (2000). Effect of breastfeeding on infant and child mortality due to infectious diseases in less developed countries: a pooled analysis. WHO Collaborative Study Team on the Role of Breastfeeding on the Prevention of Infant Mortality. Lancet.

[8] Betrán AP, De Onís M, Lauer JA, Villar J (2001). Ecological study of effect of breast feeding on infant mortality in Latin America. BMJ.

[9] Oddy WH, Sly PD, de Klerk NH, Landau LI, Kendall GE, Holt PG (2003). Breast feeding and respiratory morbidity in infancy: a birth cohort study. Arch Dis Child.

[10] Dewey KG, Cohen RJ, Brown KH, Rivera LL (2001). Effects of exclusive breastfeeding for four versus six months on maternal nutritional status and infant motor development: results of two randomized trials in Honduras. J Nutr.

[11] Giugliani ERJ (2000). Breast feeding in clinical practice. J Pediatr (Rio J).

[12] Hamill PVV, Drizd TA, Johnson CL, Reed RB, Roche AF (1977). NCHS growth curves for children: birth-18 years, United States.

[13] (1995). Word Heath Organization. Physical status: the use and interpretation of anthropometry. Report of a WHO Expert Committee.

[14] (1995). An evaluation of infant growth: the use and interpretation of anthropometry in infants. WHO Working Group on Infant Growth. Bull Word Health Organ.

[15] (1995). American Society for Parenteral and Enteral Nutrition. Definition of terms used in ASPEN. Guidelines and standards. J Parent Enter Nutr.

[16] (1999). Almeida CAN, Ricco RG, Nogueira, MPC, Del Ciampo La, Muccillo G. Evaluation of the use of the 10th percentile of weight for age as a cut point for detection of infants under nutritional risk. J Pediatr (Rio J).

[17] World Health Organization (1991). Indicators for assessing breastfeeding practices.

[18] Kramer MS, Kakuma R (2001). The optimal duration of exclusive breastfeeding: a systematic rewiew.

[19] Panich HB (2003). Respiratory syncytial virus bronchiolitis supportive care and therapies designed to overcome airway obstruction. Pediatr Infect Dis J.

[20] Jafri HS (2003). Treatment of respiratory syncytial virus: antiviral therapies. Pediatr Infect Dis J.

[21] Tepper RS, Morgan WJ, Cota K, Wright A, Taussig LM (1986). Physiologic growth and development of the lungs during the first year of life. Am Rev Respir Dis.

[22] Albernaz EP, Menezes AMB, Césae JA, Victora CG, Barros FC, Halpern R (2003). Risk factors associated with hospitalization for bronchiolitis in the post-neonatal period. Rev Saúde Pública.

[23] Rubin FM, Fischer GB (2003). Clinical and transcutaneous oxygen saturation characteristics in hospitalized infants with acute viral bronchiolitis. J Pediatr (Rio J).

[24] Pelletier DL (1995). Potentiating effects of malnutrition on child mortality: epidemiologic evidence and policy implications. Food Nutr Bull.

[25] Victora CG, Smith PG, Vaughan JP, Nobre LC, Lombardi C, Teixeira AM (1987). Evidence for protection by breast-feeding against infant deaths from infectious diseases in Brazil. Lancet.

[26] Victora CG Breast-feeding, morbidity and mortality. In: Chandra RK, editor. Proceeding of the Conference on Nutrition and Immunology St. John's, Newfoundland: Biomedical Publishers and Distributors. 1992;63–72.

[27] Bachrach VRG, Shwarz E, Bachrach LR (2003). Breastfeeding and the risk of hospitalization for respiratory disease in infancy. A meta-analysis. Arch Pediatr Adolesc Med.

[28] Pan American Health Organization (2003). Guiding principles for complementary feeding of the breastfed child.

[29] Word Heath Organization (2003). Global strategy for infant and young child feeding.

[30] Marques RFSV, Lopez FA, Braga JAP (2004). Growth of exclusively breastfed infants in the first 6 months of life. J Pediatr (Rio J).

[31] Pickering LK, American Academy of Pediatrics (2003). Respiratory syncytial virus. Red book: 2003 Report of the Committee on Infectious Diseases.

[32] Yoon PW, Black RE, Moulton YJ, Diaz A, Benguigui Y, Land S, Paganini JM, Yunes J (1997). A situação da saúde materno-infantil e suas tendências na América Latina e Caribe. Ações de saúde materno infantil a nível local segundo as Metas da Cúpula Mundial em Favor da Infância.

[33] Ferreira HS, França AOS (2002). Evolution of nutritional status in hospitalized infants J Pediatr (Rio J).

